# Clinical characteristics and prognostic factors of COVID-19 patients progression to severe: a retrospective, observational study

**DOI:** 10.18632/aging.103931

**Published:** 2020-10-14

**Authors:** Yunfei Liao, Yong Feng, Bo Wang, Hanyu Wang, Jinsha Huang, Yaxin Wu, Ziling Wu, Xiao Chen, Chao Yang, Xinqiao Fu, Hui Sun

**Affiliations:** 1 Department of Endocrinology, Union Hospital, Tongji Medical College, Huazhong University of Science and Technology, Wuhan 430022, China; 2 Department of Orthopedics, Union Hospital, Tongji Medical College, Huazhong University of Science and Technology, Wuhan 430022, China; 3 Department of Rehabilitation, Wuhan No.1 Hospital, Tongji Medical College, Huazhong University of Science and Technology, Wuhan 430022, China; 4 Department of Neurology, Union Hospital, Tongji Medical College, Huazhong University of Science and Technology, Wuhan 430022, China; 5 First Clinical College, Tongji Medical College, Huazhong University of Science and Technology, Wuhan 430030, China; 6 Department of Vascular Surgery, Union Hospital, Tongji Medical College, Huazhong University of Science and Technology, Wuhan 430022, China; 7 Outpatient Department, Union Hospital, Tongji Medical College, Huazhong University of Science and Technology, Wuhan 430022, China

**Keywords:** COVID-19, clinical characteristics, prognostic factors, nomogram

## Abstract

The outbreak of coronavirus disease 2019 (COVID-19) has become a world-wide emergency. The severity of COVID-19 is highly correlated with its mortality rate. We aimed to disclose the clinical characteristics and prognostic factors of COVID-19 patients who developed severe COVID-19. The study enrolled cases (no=1848) with mild or moderate type of COVID-19 in Fangcang shelter hospital of Jianghan. A total of 56 patients progressed from mild or moderate to severe. We used least absolute shrinkage and selection operator regression model to select prognostic factors for this model. The case-severity rate was 3.6% in the shelter hospital. They were all symptomatic at admission. Fever, cough, and fatigue were the most common symptoms. Hypertension, diabetes and coronary heart diseases were common co-morbidities. Predictors contained in the prediction nomogram included fever, distribution of peak temperature (>38°C), myalgia or arthralgia and distribution of C-reactive protein (≥10 mg per L). The distribution of peak temperature (>38°C) on set, myalgia or arthralgia and C-reactive protein (≥10 mg per L) were the prognostic factors to identify the progression of COVID-19 patients with mild or moderate type. Early attention to these risk factors will help alleviate the progress of the COVID-19.

## INTRODUCTION

Recent study has shown that the coronavirus disease 2019 (COVID-19) has the ability of human-to-human transmission and becomes a world-wide emergency [1]. The World Health Organization (WHO) has recently declared COVID-19 outbreak in several countries. Since January 2020, thousands of new patients have been diagnosed every day, which requires enormous medical resources. The surge of infections placed huge pressure on the national medical system [2].

The Fangcang shelter hospitals in Wuhan were large-scale, temporary hospitals, rapidly built by converting existing public venues, such as exhibition centers and stadiums, into health-care facilities. They were served to isolate patients with mild or moderate COVID-19 from their families and communities, while providing basic medical care, disease monitoring, food, shelter, and social activities [3]. Patients with mild or moderate COVID-19 who met additional admission criteria were isolated and treated in the Fangcang shelter hospitals, whereas patients with severe or critical COVID-19 received medical care in traditional hospitals [3–6]. Fangcang shelter hospitals provide basic medical care and monitored the progression of disease. As some patients remain experienced progression of COVID-19 or development of severe chronic diseases, they were transferred in a timely manner to the designated higher-level hospitals. The clinical characteristics of patients transferred to the designated hospital were important for the revision of admission criteria of COVID patients in Fangcang shelter hospitals.

The severity of the COVID-19 determines the fatality rate and the medical resource usage. The case-severity (mild or moderate progressing to severe case) rate was an important benefit index for therapeutic efficacy assessment in shelter hospital. Dynamic observation the risk factors of mild to severe patients is contribute to great value for early prognosis and treatment. Therefore, a retrospective review of overall medical record was performed in Fangcang shelter hospital of Jianghan, which received the largest number of patients among Fangcang shelter hospitals in Wuhan. A total of 1848 cases with mild or moderate COVID-19 were included and 521 cases transferred to the designated hospital were analyzed. The clinical characteristics and prognostic factors of patients from mild or moderate to severe were detected as well.

## RESULTS

### Outcomes and case-severity rate of patients in Fangcang shelter hospital

**Figure 1:**
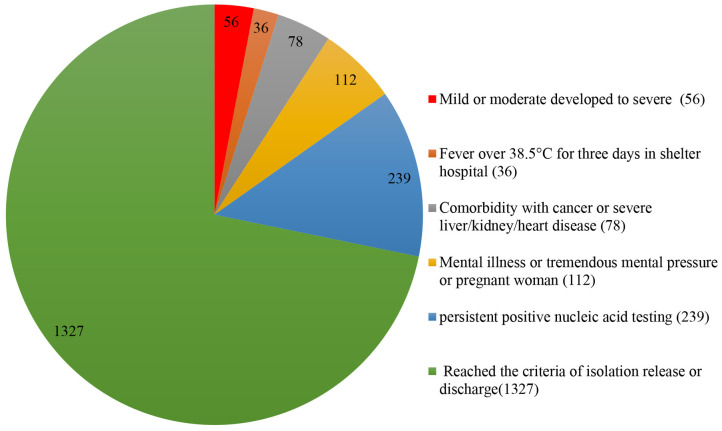
**Outcome and distribution of all enrolled patients in Jianghan Fangcang shelter hospital (1848).**

Among 1848 enrolled patients, the age range was from 15 to 81 years, and 49.0% were men. From Feb 5^th^ to Mar 9^th^ 2020, 521 (28.2%) patients were transferred to the designated hospitals for further treatment. Meanwhile, the other 1327 (71.8%) patients reached the criteria of isolation release or discharge, (Figure 1). Among 521 patients transferring to the designated hospitals, 10.7% patients with severe type from mild or moderate type (56 cases), 6.9% (36 cases) patients with body temperature more than 38.5°C for 3 days or more after treatment, 15.0% (78 cases) patients with cancer or severe liver/kidney/heart disease, 45.9% (239 cases) patients with the persistent positive nucleic acid testing after 2 weeks treatment, and 21.5% (112 cases) patients with other reasons (including new onset severe symptoms or mental illness or tremendous mental pressure or pregnant woman). The basic clinical characteristics of patients transferring to the designated hospitals were in Table 1.

**Table 1:** Clinical characteristics of patients transferring to the designated hospitals from fangcang shelter hospital.

	**Mild or moderate progressed to severe (56)**	**Fever over 38.5°C for three days in shelter hospital (36)**	**Comorbidity with cancer or severe liver/kidney/heart disease (78)**	**Mental illness, mental pressure or pregnant woman (112)**	**Persistent positive nucleic acid testing (239)**
Age	55 (48-61)	52 (44-58)	62 (52-64)	54 (48-62)	53 (45-61)
Female sex	26(46.4%)	22 (61.1%)	58 (74.4%)	47 (42.0%)	132 (55.2%)
Median incubation (onset to cabin) period	10 (8-16)	12 (6-13)	12 (10-23)	12 (7-18)	13 (9-19)
Median cabin period	4 (3-8)	7 (4-12)	17 (13-22)	16 (12-21)	18 (14-22)
Median incubation (onset to transfer) period	15 (12-23)	20 (11-25)	30 (29-37)	19 (17-26)	31 (25-37)
Fever on cabin admission					
Patients	43(76.8%)	24 (67.7%)	16 (20.5%)	52 (46.4%)	88 (36.8%)
Median temperature (of peak value)	38.2±0.71	38.8±0.47	38.1±0.39	38.2±0.68	38.2±0.53
Symptoms at onset					
Cough	34 (60.7%)	18 (50.0%)	33 (42.3%)	50 (44.6%)	88 (36.8%)
Sputum production	3 (5.4%)	1 (2.8%)	5 (6.4%)	8 (7.1%)	15 (6.3%)
Nasal congestion	4 (7.1%)	1 (2.8%)	3 (3.8%)	9 (8.0%)	13 (5.4%)
Sore throat	11(19.6%)	6 (16.7%)	10 (12.8%)	14 (12.5%)	31 (13.0%)
Shortness of breath	10(17.9%)	6 (16.7%)	10 (12.8%)	16 (14.3%)	29 (12.1%)
Stomach ache	1(1.8%)	0	1 (1.3%)	2 (1.8%)	5 (2.1%)
Chest pain	5 (8.9%)	3 (8.3%)	6 (7.7%)	8 (7.1%)	17 (7.1%)
Chest congestion	10(17.9%)	3 (8.3%)	8 (10.3%)	13 (11.6%)	32 (13.4%)
Nausea or vomiting	4 (7.1%)	1 (2.8%)	5 (6.4%)	7 (6.3%)	10 (4.2%)
Diarrhea	11(19.6%)	5 (13.9%)	20 (12.8%)	18 (16.1%)	16 (6.7%)
Myalgia or arthralgia	17 (30.4%)	9 (25.0%)	18 (25.6%)	30 (26.8%)	60 (25.1%)
Poor appetite	16 (28.6%)	9 (25.0%)	17 (21.8%)	27 (24.1%)	57 (23.8%)
Headache	2 (3.6%)	2 (5.6%)	2 (2.6%)	3 (2.7%)	11 (4.6%)
Fatigue	32 (57.1%)	20 (55.6%)	37 (47.4%)	52 (46.4%)	71 (29.7%)
Chills	5 (8.9%)	3 (8.3%)	4 (5.1%)	8 (7.1%)	18 (7.5%)
Palmic	2 (3.6%)	1 (2.8%)	1 (1.3%)	2 (1.8%)	10 (4.2%)
Perspire	1(1.8%)	0	1 (1.3%)	1 (0.9%)	3 (1.3%)
Comorbidity					
Any	23 (41.1%)	14 (38.9%)	78 (100%)	49 (43.8%)	82 (34.3%)
Chronic obstructive pulmonary disease	1 (1.8%)	1 (2.8%)	10 (12.8%)	2 (1.8%)	6 (2.5%)
Diabetes	4 (7.1%)	2 (5.6%)	16 (20.5%)	8 (7.1%)	13 (5.4%)
Hypertension	13 (23.2%)	7 (19.4%)	25 (32.1%)	27 (24.1%)	45 (18.8%)
Coronary heart disease	4 (7.1%)	2 (5.6%)	9 (11.5%)	8 (7.1%)	11 (5.3%)
Cerebrovascular disease	1 (1.8%)	1 (2.8%)	9 (11.5%)	2 (1.8%)	4 (1.7%)
Chronic renal disease	1 (1.8%)	0	9 (11.5%)	2 (1.8%)	3 (1.3%)

Data are median (IQR), n (%) or n/N (%).

The case-severity rate was identified as the proportion of mild or moderate type progressing to severe type in this study. A total of 56 patients have progressed from mild or moderate type to the severe type. The case-severity rate of COVID-19 was 3.6% (67/1848) in the Fangcang shelter hospital of Jianghan.

### Clinical characteristics of patient progression from mild or moderate type to severe type

In order to better display the clinical characteristics of mild or moderate to severe patients, 92 age-and sex-matched mild or moderate patients stayed in the Fangcang shelter hospital at the same time have been showed and compared as well. The severe patients have the following characteristics. Among 56 severe patients, 30 were male (53.6%) and 26 were female (46.4%). All the patients aged 28-73 years with an average age of 55 years (IQR 48.0-61.0). About 76.8% of those patients was between 40 to 65 years. The median incubation time (onset to severe) was 10 days (IQR 8.0-16.0), 7/56 (12.5%) less than 7 days, 34/56 (60.7%) between 7-14 days, 14/56 (25.0%) more than 14 days. Meanwhile, the median time in mobile cabin was 4 days (IQR 3.0-8.0), 12/56 (21.8%) less than 3 days, 29/56 (52.7%) between 3-7 days, 14/56 (25.5%) more than 7 days (Table 2).

**Table 2:** Clinical characteristics of the mild or moderate progressed to severe patients.

	**Mild or moderate progressed to severe (56)**	**Match mild or moderate (92)**	***P* value**
Age			
Median (IQR) — year	55 (48-61)	56 (48-62)	0.909
Distribution of age— no./total no. (%)	..	..	0.976
<40	7 (12.5%)	11 (12.0%)	..
40-65	43 (76.8%)	72 (78.3%)	..
>65	6 (10.7%)	9 (9.8%)	..
Female sex — no./total no. (%)	26(46.4%)	48 (52.2%)	0.498
Smoking history — no./total no. (%)	3 (5.4%)	5 (5.4%)	0.984
Median incubation (onset to shelter) period (IQR) — days	10 (8-16)	14 (11-19)	<0.0001
Distribution of period— no./total no. (%)	..	..	0.016*
<7days	7(12.5%)	9 (9.8%)	..
7-14days	34 (60.7%)	36 (39.1%)	..
>14days	14 (25.0%)	44 (47.8%)	..
Median cabin period (IQR) — days	4 (3-8)	17 (12-22)	<0.0001
Distribution of period — no./total no. (%)	..	..	<0.0001*
<3days	12 (21.8%)	1 (1.1%)	..
3-7days	29(52.7%)	10 (11.0%)	..
>7days	14 (25.5%)	80 (87.9%)	..
Median incubation (onset to transfer) period (IQR) — days	15 (12-23)	33 (28.5-37)	<0.0001
Distribution of period — no./total no. (%)	..	..	<0.0001*
<7days	2 (3.6%)	0	..
7-14days	24 (43.6%)	2 (2.2%)	..
>14days	29 (52.7%)	87 (97.8%)	..
Fever on cabin admission			
Patients — no./total no. (%)	43(76.8%)	24 (26.1%)	<0.0001
Mean temperature (of peak value) (SDs)— °C	38.2±0.71	37.7±0.58	0.011
Distribution of peak temperature — no./total no. (%)	..	..	0.046*
<37.3°C	4 (7.1%)	22 (23.9%)	..
37.3–38.0°C	21(37.5%)	54 (58.7%)	..
38.1–39.0°C	24(42.9%)	16 (17.4%)	..
>39.0°C	7 (12.5%)	0	..
Symptoms at onset — no. (%)			
Cough	34 (60.7%)	36 (39.1%)	0.011
Sputum production	3 (5.4%)	11 (12.0%)	0.183
Nasal congestion	4 (7.1%)	2(2.2%)	0.137
Sore throat	11(19.6%)	4(4.4%)	0.003
Shortness of breath	10(17.9%)	4(4.4%)	0.006
Stomach ache	1(1.8%)	2(2.2%)	0.871
Chest pain	5 (8.9%)	3(3.3%)	0.139
Chest congestion	10(17.9%)	13(14.1%)	0.544
Nausea or vomiting	4 (7.1%)	3(3.3%)	0.281
Diarrhea	11(19.6%)	7(7.6%)	0.03
Myalgia or arthralgia	17 (30.4%)	6(6.5%)	<0.0001
poor appetite	16 (28.6%)	6(6.5%)	<0.0001
Headache	2 (3.6%)	7(7.6%)	0.319
Fatigue	32 (57.1%)	25(27.2%)	<0.0001
Chills	5 (8.9%)	5(5.4%)	0.412
Palmic	2 (3.6%)	6(6.5%)	0.441
Perspire	1(1.8%)	0	0.198
Cough	34 (60.7%)	36 (39.1%)	0.011
Sputum production	3 (5.4%)	11 (12.0%)	0.183
Comorbidity — no. (%)			
Any	24 (42.9%)	28 (30.4%)	0.187
Chronic obstructive pulmonary disease	1 (1.8%)	2 (2.2%)	0.871
Diabetes	4 (7.1%)	3 (3.3%)	0.281
Hypertension	13 (23.2%)	17 (18.5%)	0.487
Coronary heart disease	4 (7.1%)	3 (3.3%)	0.281
Cerebrovascular disease	1 (1.8%)	3 (3.3%)	0.591
Chronic renal disease	1 (1.8%)	0	0.198

Data are mean (SDs), median (IQR), n (%), or n/N (%). P values were calculated by Student t test, Mann-Whitney U test, χ² test, or Fisher’s exact test, as appropriate. *χ² test comparing all subcategories.

The clinical manifestations at onset of those patients have been observed. There were no asymptomatic cases on admission. Besides, 43/56 (76.8%) of patients were with fever on admission, 31/56 (55.4%) of patients were once with fever over 38.0°C, 34/56 (60.7%) with cough, 11/56 (19.6%) with sore throat, 10/56 (17.9%) with shortness of breath, 17/56 (30.4%) with myalgia or arthralgia, 11/56 (19.6%) with diarrhea, 16 (28.6%) with poor appetite, 32 (57.1%) with fatigue.

There were 42.9% (24/56) of patients with comorbidity. The most common comorbidity was hypertension (23.2%), followed by diabetes (4/56, 7.1%), coronary heart diseases (4/56, 7.1%), kidney diseases (1/56, 1.8%), cerebral infarction (1/56, 1.8%) and chronic obstructive pulmonary disease (1/56, 1.8%).

At admission, lymphocytopenia was less common (in 35.7% of the patients), with a mean lymphocyte count of 1.70±0.72×10^9^ per L in this study. Most of the patients showed elevated levels of C-reactive protein (CRP) at ill onset with a median CRP of 10.12 mg per L (IQR 1.33-16.44). The CRP levels of 33.3% patients were over 10 mg/L. All the patients were examined by chest CT 6±3 days after the onset of disease. As shown in the imaging, 24/56 (42.9%) of patients were with bilateral distribution, followed by 22/56 (39.3%) with local distribution, 11/56 (19.6%) with multiple distribution, 7/56 (12.5%) with unilateral distribution. Meanwhile, ground-glass opacity (53/56, 94.6%) was the most common morphological depiction, followed by 10/56 (17.9%) patchy shadowing, 1/56 (1.8%) interstitial abnormalities (Table 3).

**Table 3:** Radiographic, laboratory findings, treatments, and clinical outcomes.

	**Mild or moderate progressed to severe (56)**	**Match mild or moderate (92)**	**P value**
**Radiologic findings**			
Abnormalities on chest CT — no./total no. (%)			
Local	22 (39.3%)	10 (10.9%)	<0.0001
Multiple	11 (19.6%)	52 (56.5%)	<0.0001
Bilateral	24 (42.9%)	72 (78.3%)	<0.0001
Unilateral	7 (12.5%)	18 (19.6%)	0.374
Patchy shadowing	10 (17.9%)	7 (7.6%)	0.034
Ground-glass opacity	53 (94.6%)	82 (89.1%)	0.432
Interstitial abnormalities	1 (1.8%)	19 (20.7%)	0.002
Laboratory findings			
White-cell count, median (IQR), × 10^9^ per L	5.75(4.66 -7.91)	5.96 (5.04-7.09)	0.848
Lymphocyte count, mean (SDs), × 10^9^ per L	1.70±0.72	1.88±0.54	0.244
Distribution of lymphocyte count— no./total no. (%)	42	90	0.468
<1.5× 10^9^ per L	15 (35.7%)	24 (26.7%)	..
≥1.5 × 10^9^ per L	27 (64.3%)	66 (73.3%)	..
Platelet count, median (IQR), × 10^9^ per L	238 (181-331)	244 (205-320)	0.724
Distribution of platelet count— no./total no. (%)	42	90	0.183
<150 × 10^9^ per L	2 (4.8%)	1 (1.1%)	..
≥150 × 10^9^ per L	40 (95.2%)	89 (98.9%)	..
Median hemoglobin (IQR) — g per dl	134 (131-147)	137 (128-144)	0.828
C-reactive protein, median (IQR), mg per L	10.12 (1.33-16.44)	1.14 (0.37-2.61)	<0.0001
Distribution — no./total no. (%)	42	90	<0.0001*
C-reactive protein ≥10 mg per L	14 (33.3%)	5 (5.6%)	..
C-reactive protein <10 mg per L	28 (66.7%)	85 (94.4%)	..
**Treatments**	42	90	..
Antibiotics, antivirus and traditional Chinese medicine	16 (38.1%)	40 (50.6%)	0.188
Antibiotics and antivirus	5 (11.9%)	3 (3.9%)	0.091
Antivirus and traditional Chinese medicine	6 (14.3%)	17 (21.8%)	0.319
**Clinical outcomes at data cutoff (2020.3.12) — no. (%)**			
Nucleic acid test	52	92	0.106
Positive	3 (5.8%)	1 (1.1%)	..
Negative	49 (94.2%)	91 (98.9%)	..
CT scan	..	..	<0.0001*
Recovery	2 (3.8%)	2 (2.2%)	..
Improved	48 (92.3%)	90 (97.8%)	..
Deterioration or new lesions	2 (3.8%)	0	..

Data are mean (SDs), median (IQR), n (%), or n/N (%). P values were calculated by Student t test, Mann-Whitney U test, χ² test, or Fisher’s exact test, as appropriate. *χ² test comparing all subcategories.

According to the symptoms and signs, patients received different treatments. There were 16/42 (38.1%) of patients with antibiotics, antivirus and traditional Chinese medicine, 5/42 (11.9%) of patients with antivirus and antibiotics, 6/42 (14.3%) of patients with antivirus and traditional Chinese medicine. There were 14 patients with no medicine records due to the short time of staying in Fangcang shelter hospital.

On March 12, those 56 patients were followed up by telephone and 4 lost. There were 5.8% (9/52) of the patients whose nucleic acid test results remain positive. As shown in the CT scan, 96.2% (50/52) of the patients were recovery or improved, only 3.8% (2/52) of the patients got deterioration or new lesions. The clinical manifestations and laboratory findings were shown in Table 3.

### The prognostic factors of patients transforming from mild or moderate type to severe type

**Figure 2:**
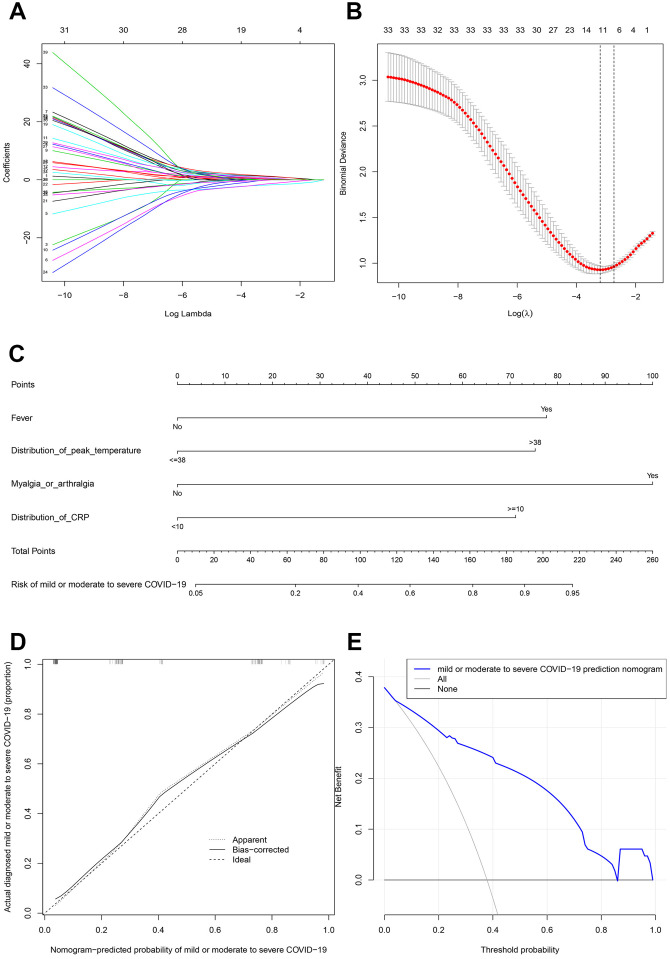
**Prognostic factor selection using the LASSO binary logistic regression model.** (**A**) LASSO coefficient profiles of the 45 variables. (**B**) Optimal parameter (lambda) selection in the LASSO model used tenfold cross-validation via minimum criteria. The partial likelihood deviance (binomial deviance) curve was plotted versus log(lambda). Dotted vertical lines were drawn at the optimal values by using the minimum criteria and the one standard error of the minimum criteria. (**C**) Developed mild or moderate to severe COVID−19 nomogram. (**D**) Calibration plot. (**E**) Decision curve.

There were 45 variables in the LASSO regression and the outcomes displayed that median incubation (onset to shelter) period (≥14 days), fever, distribution of peak temperature (>38°C), sputum production, sore throat, shortness of breath, myalgia or arthralgia, poor appetite, headache, diabetes and CRP (≥10 per L) were predictive factors for patients transforming from mild or moderate type to severe type with nonzero coefficients (Figure 2A, 2B). Ten variables mentioned above were then included in the multiple logistic regression analysis. The results demonstrated that fever, distribution of peak temperature (>38°C), myalgia or arthralgia and distribution of CRP (≥10 per L) were significantly predictive factors (Table 4). Moreover, the model that incorporated the above independent predictors was developed and presented as the nomogram (Figure 2C). The calibration curves of this nomogram showed good agreement in this cohort (Figure 2D). The C-index of the prediction nomogram was 0.888 (95% CI 0.839-0.937) for this cohort, which was confirmed to be 0.876 by bootstrapping validation. In this risk nomogram, effective performance showed a good prediction capability. Furthermore, the decision curve demonstrated that the prediction nomogram had superior standardized net benefit while the threshold probability of a patient and a doctor is 4% and 83%, respectively (Figure 2E).

**Table 4:** Risk factors associated with mild or moderate pregressed to severe transfer from fangcang shelter hospital to the designated hospital.

**Logistic Regress Variables**	***β***	**Multivariable OR (CI 95%)**	**P Value**
Median incubation (onset to shelter) period (≤14 VS ≥14 days)	-0.529	0.588 (0.158-2.076)	0.412
Fever on shelter admission (Patients — no./total no. (%))	2.599	13.459 (4.147-54.650)	<0.0001*
Distribution of peak temperature (≤38°C VS >38°C)	2.421	11.261 (3.405-43.737)	<0.0001*
Sputum production	-1.127	0.324 (0.051-1.697)	0.195
Sore throat	1.929	6.884 (0.977-77.611)	0.081
Shortness of breath	1.706	5.506 (0.798-50.485)	0.096
Myalgia or arthralgia	2.665	14.375 (3.223-83.279)	0.001*
Poor appetite	1.335	3.802 (0.785-21.792)	0.111
Headache	-2.179	0.113 (0.007-1.082)	0.083
Diabetes	1.273	3.570 (0.298-48.145)	0.318
Distribution of CRP (<10 VS ≥10 per L)	2.254	9.530 (2.145-49.634)	0.004*

*β* is the regression coefficient. OR=odds ratio. CI, confidence interval. *Statistically significant.

Furthermore, by utilizing receiver operating characteristic (ROC) analysis, we compared the predictive value of our prediction model for incidence of severe illness with that of the MuLBSTA, CURB-6 and NLR models. The analysis revealed that our prediction nomogram had the highest area under curve (AUC) (0.902) than the other three models (Supplementary Figure 1).

## DISCUSSION

It is the first time to observed dynamically and comprehensively disclose the clinical characteristics and prognostic factors of COVID-19 patient progression to severe. The number of patients with severe type determines the final mortality rate of COVID-19. In this study, the case-severity rate was observed with a relatively large prospective cohort, which might be a valuable complement to the characteristics of COVID-19. In the Fangcang shelter hospital of Jianghan, about 3.0% of the patients transformed to severe, which was significantly lower than the 14% cases classified as severe or critical in the spectrum of COVID-19 disease [7, 8]. The most common symptom at admission was fever and 76.8% (43/56 cases) mild to severe patients got fever on cabin admission. However, some patients with Covid-19 did not have fever abnormalities on initial presentation, which has complicated the diagnosis [9]. In this study, 55.4% patients were with peek temperature more than 38.0°C, while 12.5% of patients were once with fever over 39.0°C. High fever was associated with the development of severity and critical death [10–12]. Therefore, keep vigilance of those mild patients whose peak temperature over 38.0°C. They were quickly transferred to the designated higher-level hospitals once the blood oxygen saturation of those patients was less than 93% in Fangcang shelter hospital of Jianghan.

For more specialized monitoring, chest imaging and laboratory services were applied in the Fangcang shelter hospitals. Ground-glass opacity (53/56, 94.6%) was the most common morphological depiction in CT scan on admission. However, only 19.6% of patients showed multiple lesions, 42.9 % patients presented bilateral lesions. Compared with mild patients, most severe patients took CT scan in this study within seven days. The lesions that were present in asymptomatic individuals progressed to bilateral diffuse disease with consolidation around day 10 after the symptom onset [13–15]. The predominant CT pattern was unilateral and multifocal ground-glass opacities in early stage, then lesions quickly evolved to bilateral, diffuse ground-glass opacity in later stage [13]. However, those characteristics were not consistent with what we had expected in this study. Therefore, the value of lung CT in determining the prognosis of mild COVID-19 patients still needs further research. Meanwhile, most of the patients had elevated levels of CRP at ill onset with 33.3% over 10 mg/L. Similarly, compared to mild or moderate cases, severe cases more frequently had higher levels of CRP [9, 16, 17]. Therefore, imaging and laboratory results could contribute to make the quick decision of transferring to the designated hospitals.

Previously, older age (over 65 years) was associated with higher odds of progression to severity of COVID-19, which also has been reported as an important independent predictor of mortality in SARS and MERS [18–20]. In order to better display the clinical characteristics of mild or moderate to severe patients below 65 years old, 92 age-and sex-matched mild or moderate patients stayed in the Fangcang shelter hospital at the same time. In this study, several factors in adults who were hospitalized in Fangcang shelter hospital were associated with mild progressed to severe COVID-19. In particular, patients aged 40-65 years constituted the highest proportion within the severe group in this study. It had reported that 75% of COVID-19 death cases previously suffered 1-2 underlying diseases, a majority of which were diabetes and cardiovascular diseases [21]. In line with above evidence, our study also found that 42.9% of the mild to severe patients had 1-2 basic diseases, such as cardiovascular diseases, cerebrovascular diseases and endocrine system diseases.

Notably, several clinical manifestations were identified as prognostic factors for progression from mild to severe in the univariate logistic regression analysis. We found that myalgia or arthralgia on admission was associated with increased odds of mild to severe (Table 4). Furthermore, 30.4% patients had the myalgia or arthralgia. Less common symptoms include poor appetite, sore throat and shortness of breath. However, respiratory system affection remained as the primary symptom [9, 22, 23]. Overall, onset of fever and myalgia or arthralgia symptoms should be closely monitored among cabin hospital, more attention should also be paid to patients on those isolation patients at home.

In this study, we developed a prediction nomogram included fever, distribution of peak temperature (>38°C), myalgia or arthralgia and distribution of C-reactive protein (≥10 per L) for patients with mild or moderate to severe COVID−19. Previous studies suggested that both MuLBSTA score and CURB-65 score were widely used to assess the mortality of pneumonia [24–26]. Moreover, a recent study had confirmed that neutrophil-to-lymphocyte ratio (NLR) could predicts severe illness patients with COVID−19 [27]. However, compared with the above three models, our prediction nomogram model seemed to have a higher C-index and AUC than them. Additionally, our model seems to be more clinical significance. Because we can predict the incidence of patients with COVID-19 from mild to severe. And the other three models mainly predict the mortality rate. Therefore, our model can play a warning role in the early stages of disease. According to the results, we suggested that patients with fever (peak temperature over 38.0°C), myalgia or arthralgia and CRP more than 10 per L should be vigilant by doctors and nurses in Fangcang shelter hospital.

There were some limitations in this study. Firstly, due to limited medical resources in Fangcang shelter hospital, not all laboratory tests have been performed, such as lactate dehydrogenase, IL-6, and serum ferritin. Secondly, the study was limited to the patients with mild or moderate infection in a single center study. Thirdly, duo to the shortage of medical resource in Wuhan, we could not track the patients who developed severe COVID-19 after transfer point in Fangcang shelter hospital. Thus, the result of 3.6% severe progression rate might be inaccurate. However, the study population is representative of cases mild developed to severe in Wuhan. To the best of our knowledge, this is the largest retrospective cohort study to observed dynamically and comprehensively disclose the clinical characteristics and risk factors for developed COVID-19 patients to severe type. The distribution of peak temperature (>38°C) on set, myalgia or arthralgia and C-reactive protein (≥10 mg per L) were the prognostic factors to identify the progression of COVID-19 patients with mild or moderate type. Early intervention in these risk factors may effectively control the development of the COVID-19. Overall, Fangcang shelter hospitals had substantially reduced the time from the onset of severe symptoms to admission to the designated hospital, compared with the alternative of home isolation [28, 29]. We believe that the information in the article will provide valuable data for other countries facing the COVID-19 pandemic, when they are establishing the national public health emergency management for COVID-19.

## MATERIALS AND METHODS

### Patient enrollment

The Jianghan Fangcang shelter hospital opened on the 5^th^ Feb and closed on the 9^th^ Mar 2020. During this period, a total of 1848 cases with COVID-19 were enrolled in Jianghan Fangcang shelter hospital of Wuhan from Feb 5^th^ to Mar 9^th^, 2020. The admission criteria of Fangcang shelter hospital were COVID-19 patients with mild or moderate type. Diagnosis of COVID-19 was based on the National health commission (NHC) of the People’s Republic of China [30]. The clinical classifications are as follows: (1) mild, the clinical symptoms are mild and no pneumonia manifestation can be found in imaging; (2) moderate, patients have symptoms like fever and respiratory tract symptoms, etc. and pneumonia manifestation can be seen in imaging; (3) severe, meet any of the following conditions: a) respiratory distress, respiratory rate≥30 breaths / min; b) the oxygen saturation ≤ 93% at a rest state; c) arterial partial pressure of oxygen/ oxygen concentration (FiO2) ≤ 300mmHg (1mmHg = 0.133kPa); d) pulmonary imaging with >50% lesions progression within 24 to 48 hours. The release of isolation and discharge standards are as follows: (1) with normal body temperature for more than 3 days; (2) with significantly recovered respiratory symptoms; (3) lung imaging shows obvious absorption and recovery of acute exudative lesion; (4) with negative results of the nucleic acid tests of respiratory pathogens for consecutive two times (sampling interval at least 1 day). Only reaching above four standards can these patients be released.

This study was approved by the Research Ethics Commission of Wuhan Union Hospital ([2020]0038) and informed consent was waived by the Ethics Commission of Wuhan Union Hospital for emerging infectious diseases.

### Baseline data collection

Before admission into Fangcang shelter hospital, all suspected patients of COVID-2019 were taken upper respiratory throat swab samples. Chest CT scan was performed as well. Clinical and laboratory findings were recorded and carefully checked. Laboratory tests included biochemical Indicators, blood routine, and C-reactive protein (CRP, normal range 0-4 mg per L). Epidemiological history, comorbidity, vital signs, symptoms and signs were recorded in detail. Patients meeting the diagnosis of mild or moderate type were admitted to the Fangcang shelter hospital. Two reviewers (HW, JH) independently checked all collected data. In case of disagreement among two reviewers, consensus was conducted by a third reviewer (YW).

### Follow-up

During the following days in Fangcang shelter hospital, the patients were re-examined for laboratory and imaging examination, and recorded symptoms, signs, treatments and outcome events. The throat swab specimens of RT-PCR test and chest CT scan were performed according to the symptoms and signs. After admission, all patients were given antiviral therapy (e.g., abidor hydrochloride) and other individualized treatments (such as antibiotics, antihypertensive and hypoglycemic therapy) according to the doctor's advice and NHC's interim guidelines [7]. The clinical outcomes of patients in the Fangcang shelter hospital were divided into three ways. They were the patients transferred to the designated hospitals for further treatments, the patients reaching the criteria of isolation release, and the patients kept treatment in the mobile cabin.

### COVID-19 nucleic acid detection and chest CT scan

Throat swab samples were stored in virus transport medium and transported to Wuhan Union Hospital for laboratory diagnosis. Throat swab specimens of all patients were subject to real time PCR tests by amplifying ORF1ab gene and N gene of SARS-CoV-2 (BioGerm, Shanghai, China). The CT examinations were carried out with a 16-row multidetector CT scanner (μCT550, Shanghai LianYing medical technology co., LTD) using the following parameters: detector collimation widths 64 ×0.6 mm, 128×0.6 mm, 64×0.6 mm, and 64×0.6 mm; and tube voltage 120 kV.

### Statistical analysis

Continuous variables were represented by mean (standard deviations, SDs) or median (interquartile range, IQR) as appropriate, categorical variables were described as number (%). Significant differences between the 2 groups (mild patients and mild to severe patients) were compared by Student *t* test, Mann-Whitney U test, chi-square or Fischer exact test where appropriate. In addition, we also used least absolute shrinkage and selection operator (LASSO) method to select the optimal predictive features in risk factors from the patients with COVID-19, and features with nonzero coefficients were selected in the LASSO model. Then, multivariable logistic regression model was used to build a predicting model by incorporating the features selected in the LASSO regression model. The variables with the *P*-value less than 0.05 were included in this model. The results were present as odds ratio (OR) [95% confidence interval]. Because all tests were two-sided, *P* value less than 0.05 was considered statistically significant. Analyses were performed using SPSS 20.0 statistical package and R version 3.6.0 was used to build nomogram, calibration and decision curve.

## Supplementary Material

Supplementary Figure 1
