# Supplementary material for: Clinical characteristics and prognostic factors of COVID-19 patients progression to severe: a retrospective, observational study
Source: Aging (Albany NY). 2020 Oct 14;12(19):18853–65. doi: 10.18632/aging.103931 (PMC7732300; doi:10.18632/aging.103931)
Supplement: Supplementary Figure 1 [file aging-12-103931-s001..pdf]

SUPPLEMENTARY FIGURE

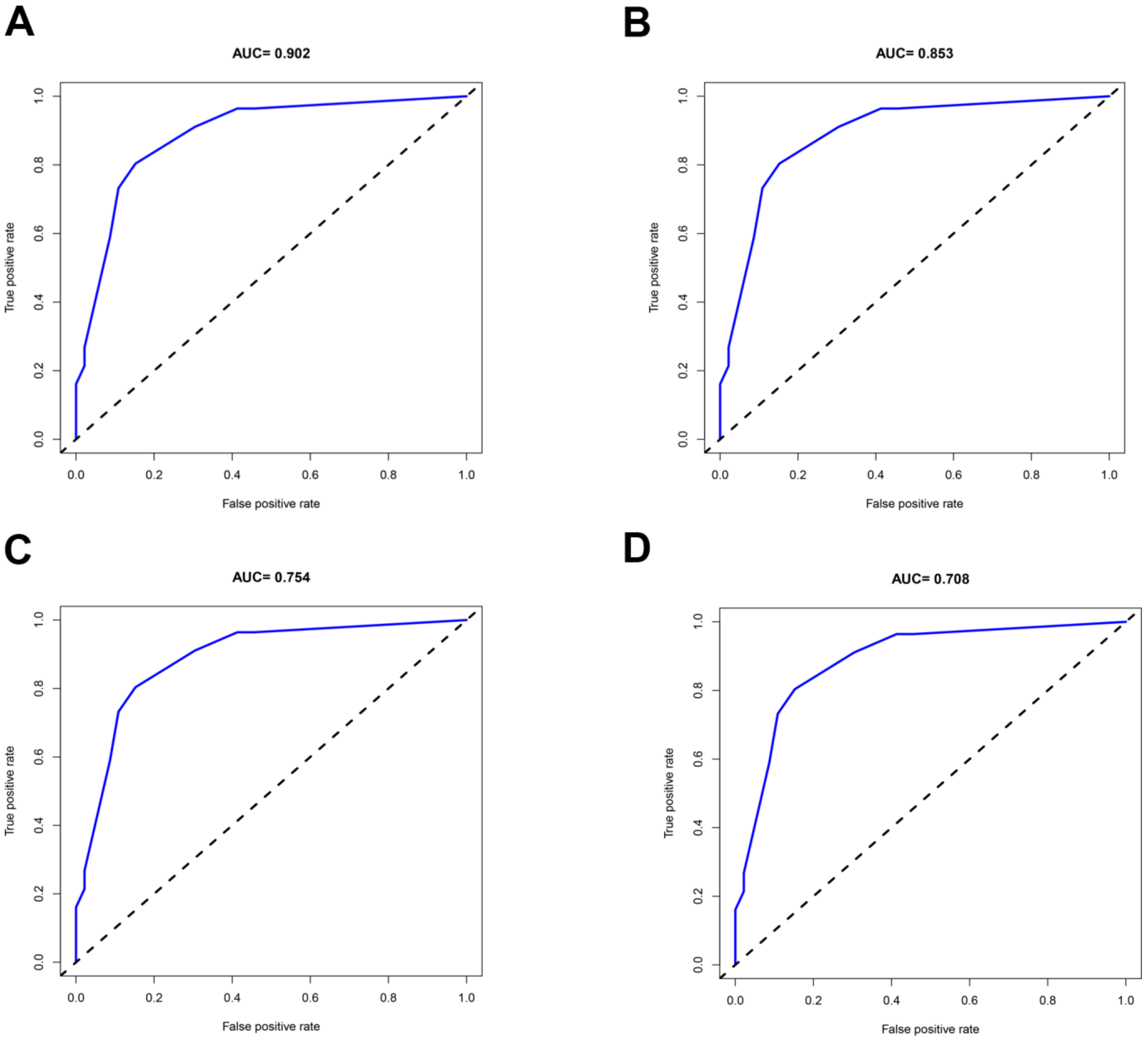

Supplementary Figure 1. Predictive value of four different models. Our prediction model (**A**); NLR (**B**), MuLBSTA (**C**) and CURB-65 (**D**).
